# Comparison between pulse wave velocities measured using Complior and measured using Biopac

**DOI:** 10.1007/s10877-018-0165-9

**Published:** 2018-06-06

**Authors:** Marit H. N. van Velzen, Robert Jan Stolker, Arjo J. Loeve, Sjoerd P. Niehof, Egbert G. Mik

**Affiliations:** 1 Laboratory of Experimental Anesthesiology, Department of Anesthesiology, Erasmus University Medical Center, Rotterdam, The Netherlands; 2 Department of BioMechanical Engineering, Faculty 3mE, Delft University of Technology, Delft, The Netherlands; 3 Department of Medical Information Communication Technology MICT, Jeroen Bosch Ziekenhuis, PO Box 90153, 5200 ME ’s Hertogenbosch, The Netherlands

**Keywords:** Arterial stiffness, Pulse wave velocity, Cardiovascular disease, Non-invasive

## Abstract

Arterial stiffness is a reliable prognostic parameter for cardiovascular diseases. The effect of change in arterial stiffness can be measured by the change of the pulse wave velocity (PWV). The Complior system is widely used to measure PWV between the carotid and radial arteries by means of piezoelectric clips placed around the neck and the wrist. The Biopac system is an easier to use alternative that uses ECG and simple optical sensors to measure the PWV between the heart and the fingertips, and thus extends a bit more to the peripheral vasculature compared to the Complior system. The goal of this study was to test under various conditions to what extent these systems provide comparable and correlating values. 25 Healthy volunteers, 20–30 years old, were measured in four sequential position: sitting, lying, standing and sitting. The results showed that the Biopac system measured consistently and significantly lower PWV values than the Complior system, for all positions. Correlation values and Bland–Altman plots showed that despite the difference in PWV magnitudes obtained by the two systems the measurements did agree well. Which implies that as long as the differences in PWV magnitudes are taken into account, either system could be used to measure PWV changes over time. However, when basing diagnosis on absolute PWV values, one should be very much aware of how the PWV was measured and with what system.

## Introduction

Arterial stiffness is a reliable prognostic parameter for cardiovascular morbidity and mortality in adults. In particular, this is the case in patients with renal disease, diabetes mellitus or hypertension and in elderly patients [1–4]. Cardiovascular disease (CVD) is worldwide the number one cause of death. Smoking, unhealthy diet, physical inactivity and harmful use of alcohol are the most important behavioural risk factors of CVD. These behavioural risks may lead to hypertension, diabetes, obesity, heart failure, or atherosclerosis. Most of these phenomena are related to an increase in arterial stiffness.

Arterial stiffness is a measure of the capability of an artery to expand and contract in response to local blood pressure changes and is the inverse of arterial compliance. The compliance, and therefore the volume change in response to a blood pressure change, in a stiff vessel is reduced compared to a healthy vessel. The effect of reduced compliance is a decreased propagation time of pressure pulse waves (PWs) through the vessels and thus an increase of the velocity of the PW. The relationship between this pulse wave velocity (PWV) and the compliance of the vessel wall is described in the MoensKorteweg equation [5]:

1 $$PWV=\sqrt {\frac{{{E_{inc}} \cdot h}}{{2r \cdot \rho }}} ,$$

where *E*_*inc*_ is the incremental elastic modulus, *h* is the wall thickness, and *r* the radius of the vessel. The symbol ρ represents the density of blood.

PWV measurements are widely used as an index of arterial stiffness [6] and for the evaluation of cardiovascular risk. PWV measurements are generally simple, accurate and highly reproducible [7, 8]. In clinical practice, several invasive and non-invasive measurement techniques are readily available to measure PWV. Two of such techniques, equally often used in clinical practice by the Erasmus Medical Centre in Rotterdam, the Netherlands, are the Complior (Alam Medical, Vincennes, France), using piezoelectric sensors [9], and the Biopac (Biopac Systems, Inc, USA), using a photoplethysmography (PPG) sensor and ECG. These techniques are generally used to non-invasively measure the PWV in the big arteries over a long trajectory.

The Complior system is used to measure PWV between the carotid and radial arteries by means of piezoelectric-clips placed around the neck and the wrist. The Biopac system is an easier-to-use alternative to the Complior system, but it measures the PWV between the heart and the fingertips, and thus extends a bit more to the peripheral vasculature. One may expect that the two systems show good agreement, because the majority of the trajectory (sternoclavicular joint to wrist) of the arterial trajectories over which the Biopac and Complior systems measure PWV are identical. However, the trajectory for the Biopac system additionally includes the wrist-fingertip vasculature. Furthermore, the Biopac includes the heart-sternoclavicular trajectory, whereas with the Complior one takes the sternoclavicular-carotid trajectory as an approximation for the heart-sternoclavicular trajectory.

While both systems are supposed to measure or approximate a PWV value for the more or less the same trajectory from the heart to the hand, the potentially differing physiological responses of the carotid and the peripheral arteries may cause different PWV measurement outcomes. The baroreceptor reflex is one of the body’s homeostatic mechanisms that helps to maintain blood pressure at nearly constant levels [10] by detecting blood pressure using the baroreceptors located in the walls of the carotid arteries. If one suddenly rises from a lying position, gravity pulls the blood in the direction of the legs, which could endanger the blood flow to the brain. As a response, the baroreceptor reflex causes the peripheral veins to be squeezed and the carotid arteries to be widened to aid the blood flow to the brain. Therefore one may expect that depending on the measurement situations the PWV change measured with the Complior system will oppose the PWV measured with the Biopac system if the baroreceptor reflex is invoked.

So while both systems are aimed at providing a similar measure of vascular condition and while the trajectories over which they measure PWV largely overlap, there are various reasons why it is unclear whether they will provide similar measurement outcomes. To the best of our knowledge, there are no reports about the agreement between PWV values measured using the Complior system or the Biopac system. Yet, in clinical practice it is crucial to know whether using different devices for the same purpose provides the same outcome. One would never accept it if measuring a heart rate using ECG versus using a pulse oximeter on the finger would provide a 30 bpm difference. Therefore, the goal of this health technology assessment was to test under various conditions to what extent these systems provide comparable values and to what extent these values correlate.

## Method

### Study population

Twenty-five healthy volunteers, 20–30 years old, without any known history of atherosclerosis associated diseases (such as diabetes mellitus, hypertension, coronary artery disease, stroke, renal disorder), or injuries at the upper limbs were included in this study after obtaining written informed consent from the subject. This study was approved by the medical ethics committee of Erasmus University Medical Center Rotterdam, The Netherlands (MEC-2012-139).

### Protocol

The transit time of a PW traveling from within the heart to easily accessible locations, such as the extremities or the neck, consists of two components: the PW propagationtime from the heart through the artery to the PW measurement location, and the isometric contraction time of the heart (pre-ejection period, PEP). The PEP is known to vary with cardiac preload and heart rate [11–13]. Therefore, all measurements were conducted in a quiet room under tranquil conditions at a room temperature of 22.4 °C (SD 0.5 °C). To further minimize any influences of a varying PEP or cardiac output during the measurement, the subjects were instructed not to talk or move during the measurement for each position. Because caffeine, tobacco and alcohol influence the heart rate and cardiac output, all subjects were asked to not take any caffeine, tobacco or alcohol for at least 3 h prior to the experiment.

The measurements were conducted 4 times for each subject, each time in a different body position. In the first position, the subject sat on a chair with both hands resting on a table. In the second position, the subject lied on a bed with both arms and hands resting along his/her body. In the third position, the subject stood upright with both hands hanging down along his/her body. The fourth position was the same as the first position, to check if the PWV-value was reproducible.

Before the start of each measurement in each position the subject was kept at rest for 60 s in the requested position. The PWV values were recorded with both of the tested systems simultaneously during the entire measurement, which lasted up to 60 s.

### Pulse wave velocity measurements and analyses

**Fig. 1:**
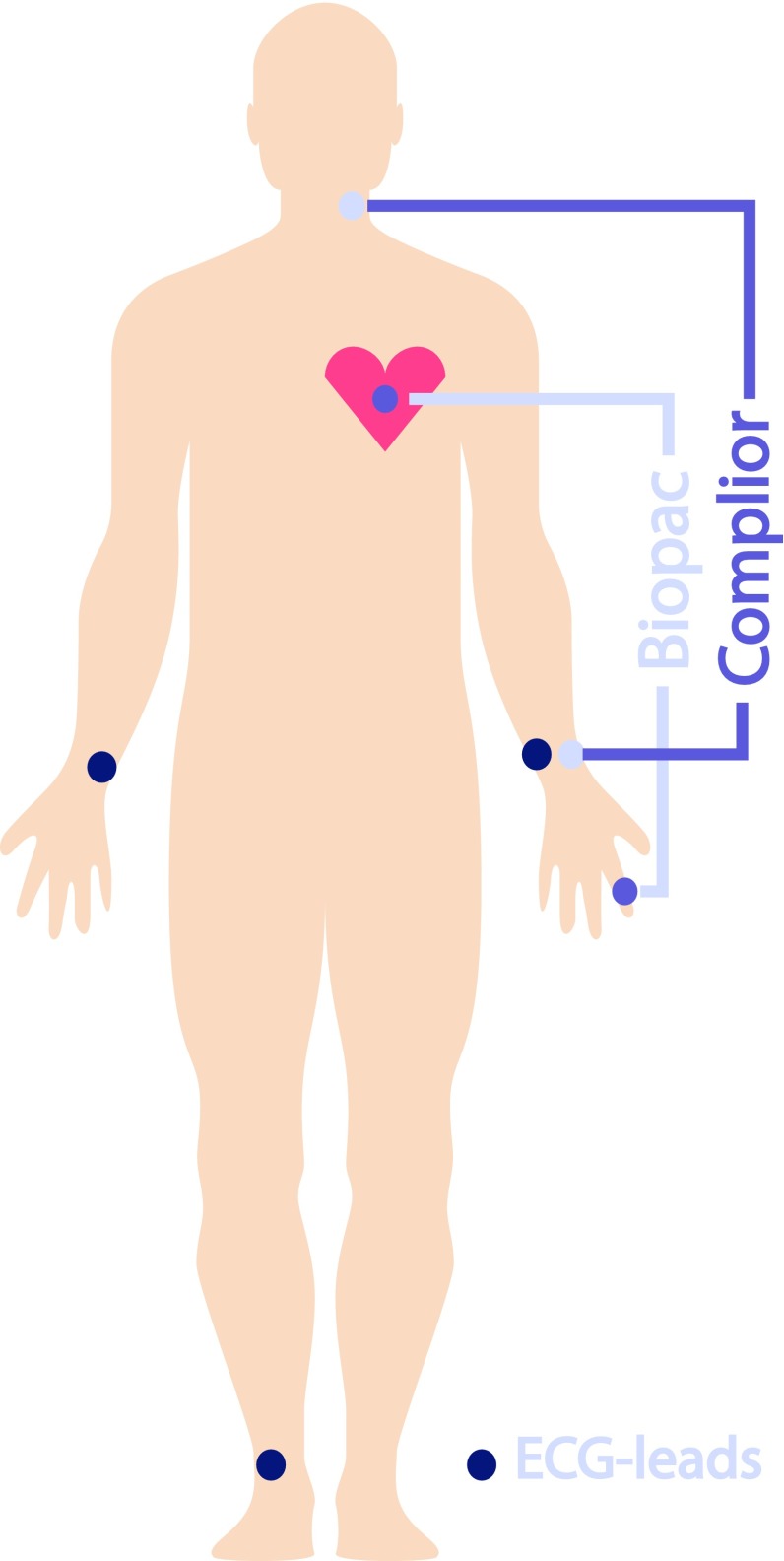
Schematic view of placement of the both measurement systems

The PWV was measured between the carotid and radial arteries using the Complior system (Alam Medical, Vincennes, France) and between the carotid artery and the left index finger tip using ECG and PPG (described below). The Complior system measures the PWV using piezoelectric sensors. For measuring the PWs on the carotid artery, a clip containing a piezoelectric sensor was placed on the left side of the neck. For measuring the PWs on the radial artery, a clip containing a piezoelectric sensor was placed on the left wrist (see Fig. 1). Both sensor signals were recorded by the “Complior SP” software. The distance between the sensors, measured in a straight line from the sternoclavicular joint to the styloid process of the radius, was used to approximate the arterial distance travelled by the PWs. Using the “Complior SP” software, the foot of the PW measured at both locations was used to calculate the mean PWV once per 5 s.

The system used for measuring the PWV between the carotid artery and the left index finger tip consisted of a measuring device and analysis software. The measurement device contained one PPG-sensor (TSD200 with the PPG100C amplifier, Biopac Systems, Inc, Goleta, USA), positioned on the left index finger, and three external ECG-leads (ECG100C amplifier, Biopac Systems, Inc, Goleta, USA) (see Fig. 1). The three ECG-leads were placed on the subject’s both wrists and right ankle. The PPG- and ECG- signals were simultaneously converted to digital signals using AcqKnowledge version 3.7.3 software (Biopac Systems, Inc, Goleta, USA), at a sampling frequency of 2 kHz. The PPG-signal was filtered with a fourth-order low pass Butterworth filter with a cut-off frequency of 9 Hz using Matlab R2010a (The MathWorks, Inc., Matick, MA, USA). The PWV was determined by dividing the distance between the PPG-sensor on the left index finger and the sternoclavicular joint (*D*) by the calculated time-difference between the time instance of the R-peak of the ECG $\left( {{t_{EC{G_{R - peak}}}}\left( n \right)} \right)$ and the foot of the PW measured at the left index finger tip $\left( {{t_{PP{G_{foot}}}}(n)} \right)$:

2 $$PW{V_{biopac}}(n)=\frac{D}{{{t_{PP{G_{foot}}}}(n) - {t_{EC{G_{R - peak}}}}(n)}},$$

where *n* is the sequence number of the heartbeats. The R-peaks in the ECG were found using the off-the-shelf Matlab function ‘R-peakdetect’ [14]. The maximum of the second derivative of each PW was taken as the foot of the PW (PPG_foot_) and the corresponding time was indicated as $\left( {{t_{PP{G_{foot}}}}(n)} \right)$ [15]. The utilized PPG-sensor was quite sensitive for motion and positioning artefacts, which sometimes distorted the shapes of the PWs in a way that they were rendered unsuitable for further analysis. Therefore, a custom-made Matlab algorithm, called ‘7Step PW-Filter’, was implemented in the data analysis to filter out any PWs that strongly deviated in shape from a suitable PW [16] .If more than 50% of the PWs were filtered out for being too distorted, the data-set was excluded from further analysis.

### Statistical analysis

The mean PWVs +/− standard deviation (SD) over 60 s were calculated for each measurement technique for each body position. The Shapiro–Wilk test was used to check if the data was normally distributed. PWV values obtained using the Complior and Biopac system were compared using a paired samples *t*-test and a Bland–Altman plot was used to analyse the agreement between the two different PWV measurement techniques. Correlation between both sitting positions (Position 1 and Position 4) were analysed using a Pearson Correlation test. A significance level of *p*-value < 0.05 was used. A repeated measurement ANOVA with GreenhouseGeisser correction and a post hoc test with Bonferroni correction was used to test for any effects of the repeated measurements. All statistical analyses were performed using SPSS version 22.0 (SPSS, Inc., Chicago, IL, USA).

## Results

Twenty-five subjects, (11 male, 14 female) were included in this study. The data of one male and one female subject were excluded from the analysis because there was too much noise in the signals to obtain any usable PWs. Table 1 presents the characteristic of the remaining study population. For positions 1, 2 and 4 (sitting 1, lying, sitting 2) the ‘7Step PW-Filter’ filtered out none of the PWs. For Position 3 (standing) there were 8 datasets with over 50% unsuitable PWs, which were therefore excluded from further analysis. In the remaining 15 datasets, the median percentage of unsuitable PWs that were filtered out was 2.2% (Q_1_ = 0% and Q_3_ = 36.6%).

**Table 1:** Characteristics of the study population

Variable	Mean ± SD (n = 23)
Gender (m/f)	10/13
Age (years)	25 ± 3
Weight (kg)	72 ± 9
Height (m)	1.77 ± 0.08
Body mass index (kg/m^2^)	23.03 ± 2.72
Blood pressure (mmHg)	
Systolic	127 ± 11
Diastolic	80 ± 9
Heart rate (bpm)	77 ± 14
Smoker, yes (%)	2 [8, 33]
Distance from sternoclavicular to wrist (cm)	69 ± 3
Distance from wrist to fingertip (cm)	17 ± 1

The means and SDs for the PWV_complior_ and PWV_biopac_ for the four different positions are listed in Table 2, as well as the results of the paired samples *t*-test. Significant differences were found for each position between PWV_complior_ and PWV_biopac_.

**Table 2:** Mean and standard deviation of the PWV

Position	PWV_complior_ (m/s)	PWV_biopac_ (m/s)	Paired sampled *t*-test
Sitting 1	10.2 ± 1.4	3.0 ± 0.2	*t*(21) = − 24.442, *p* < 0.001
Lying	9.3 ± 1.6	3.1 ± 0.2	*t*(19) = − 18.654, *p* < 0.001
Standing	9.8 ± 2.2	3.2 ± 0.2	*t*(13) = − 16.178, *p* < 0.001
Sitting 2	10.2 ± 1.1	3.0 ± 0.2	*t*(21) = − 31.704, *p* < 0.001

**Fig. 2:**
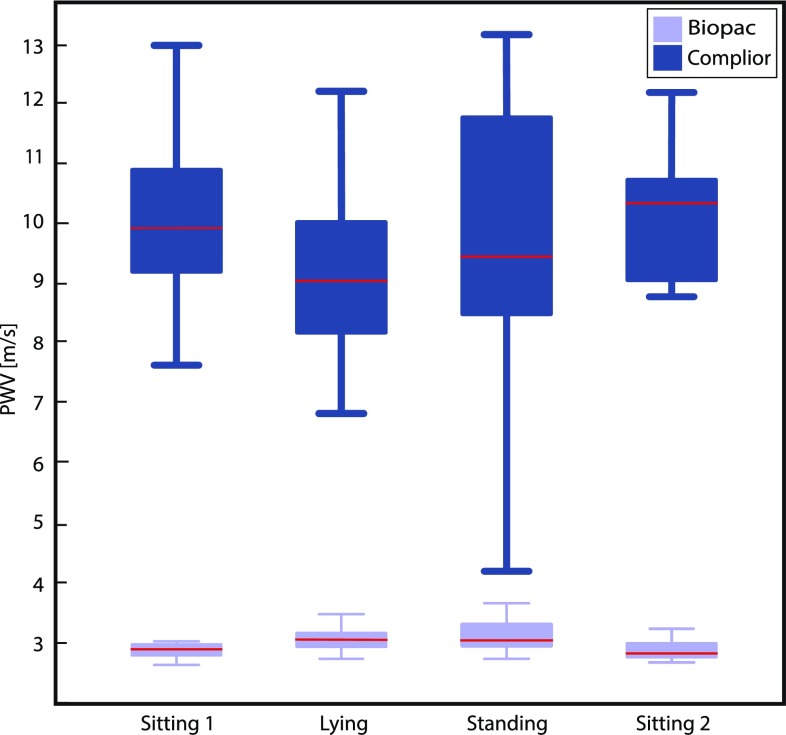
Boxplot of the PWV_biopac_ and PWV_complior_, with the median as red line and the minimum and maximum value

Figure 2 shows a boxplot of PWV_complior_ and PWV_biopac_ for each position. The data consistently showed that PWV_biopac_ was much lower than PWV_complior_.

**Fig. 3:**
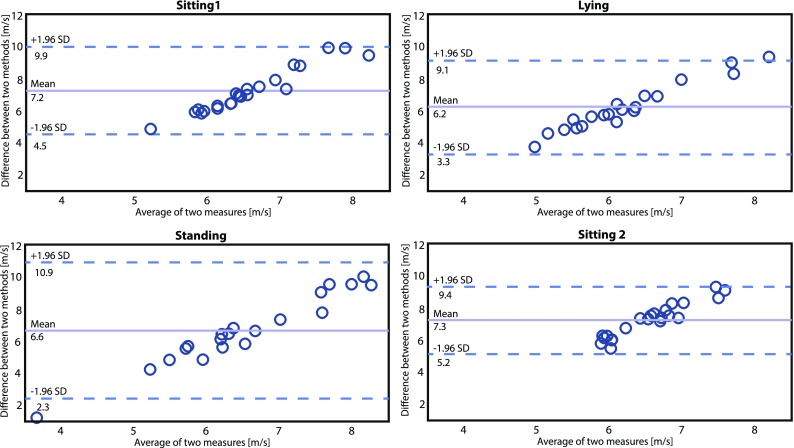
Bland–Altman plots of PWV_complior_ and PWV_biopac_ by the four positions. The dotted lines represent the 95% limits of agreement and the straight mean difference (bias) between PWV_complior_ and PWV_biopac_

Figure 3 and Table 3 present the Bland–Altman value and plot showing good agreement between the two PWV measurement techniques.

**Table 3:** Bland–Altman

Position	BIAS ± CI (m/s)	% of overall mean PWV (%)
Sitting 1	7.2 ± 2.7	37.6
Lying	6.2 ± 2.9	47.0
Standing	6.6 ± 4.3	64.4
Sitting 2	7.3 ± 2.1	29.0

The Pearson correlation between sitting Positions 1 and 4 proved to be high and significant for PWV_biopac_ (0.778, *p* < 0.001). For PWV_complior_ there was no significant correlation (0.141, *p* = 0.541).

The repeated measures ANOVA indicated that the four body positions were rated equally [F(3,45) = 2.47, *p* = 0.074], for the Complior. For the Biopac, the four body positions were not rated equally [F(3,39) = 13,1, *p* = 0.000]. The post hoc tests of the Biopac-data revealed that Position 2 (lying) and Position 3 (standing) resulted in significantly higher PWV compared to Position 1 (sitting 1) (*p* = 0.000, *p* = 0.025, respectively). There was no significantly difference between Position 2 (lying) and Position 3 (standing) (*p* = 1.000).

## Discussion

This study compared PWV values measured over the carotid-radial artery trajectory using the Complior system with PWV values measured between the R-peak of the ECG and the arrival of the PW in the left index finger tip using the Biopac system in healthy volunteers in three body positions: sitting, lying and standing. The PWV_biopac_ values were considerably lower than the PWV_complior_-values, and this effect persisted in each position. This absolute difference might be explained for a minor part by the fact that PWV_biopac_ includes the PEP, whereas PWV_complior_ does not. However, the PEP could account for no more than 1 m/s of the PWV_biopac_. Therefore, the large absolute difference between PWV_biopac_ and PWV_complior_ may more likely be explained by the difference in vessel compliance between the two trajectories. More peripheral vessels are narrower, thinner walled and more compliant. Because the more peripherally measured PWV_biopac_ showed to be lower, it is hypothesized that the reduced vessel stiffness and wall thickness (both reducing PWV) outweigh the reduced vascular radii (which would increase the PWV). However, the PWV_complior_-values agree with values reported in other studies. The mean PWV_complior_-values (10.2 ± 1.4 m/s) were in the same range as those reported by Rajzer et al. [17] (10.1 ± 1.7 m/s). Although Raizer et al. measured PWV over the carotid-femoral trajectory, it is expected that PWVs over that trajectory will be similar to those in the carotid-radial trajectory, because of similar lengths and because the effects of differences in vessel radii and wall thicknesses are likely to cancel each other out, according to Eq. 1. Furthermore, the current results also agree with those of McEleavy, who measured a PWV of 9.01 ± 1.2 m/s in the carotid-radial trajectory [18]. For the PWV_biopac_ no other studies reporting PWV between the heart and a fingertip were found, however the time between the R-peak of the ECG and arrival of the foot of the PW at the fingertip (called pulse transit time) (283 ± 21 ms) was in the same range as reported previously by van Velzen et al. (273 ± 20 ms) [16] and by Kortekaas et al. (271 ± 28 ms) [13].

Table 2 shows that the PWV_biopac_ was consistently and significantly lower than PWV_complior_ for all positions (1–4). However, the Bland–Altman plots (Fig. 3) show that the bias is small and the values are scattered around the mean, leading to the conclusion that there is a good agreement between the PWV_complior_ and PWV_biopac_ values, but they simply differ in magnitude.

The PWV was slightly, but significantly higher when lying or standing as compared to sitting (with no significant difference between lying and standing), when measured by the Biopac system over the heart-fingertip trajectory. PWV may increase when vessels become stiffer and narrower due to contraction, but although this effect could be induced when standing up, it is less likely to happen when lying down. There was no significant effect of the different positions for the PWV_complior_, which suggests that the PWV_complior_ is less suitable to detect such small PWV changes or it is less sensitive to changing positions. The Pearson correlation results show much better agreement between repetitions of the sitting position at different moments for the Biopac system than for the Complior system. This suggests that when doing longitudinal PWV measurements, the Biopac system should be preferred, provided that the same position is consistently used during successive measurements.

When using the Biopac system, the measured PWV includes the PEP. The PEP is known to vary during position changes. PEP variations caused by subject movement or stress were avoided during the current study. Kortekaas et al. showed a variability of the PEP in healthy volunteers in rest of 58.5 ± 13.0 ms [13]. Over the distances travelled by the PWs in the current study, these PEP durations could account for no more than 1 m/s of the PWV_biopac_. Consequently, PEP variations are unlikely to explain any variations in this study.

Limitations of the two tested techniques include the challenge of accurately positioning the sensors, and the discrepancy between the measured distance between the sensors and the actual path length travelled by the PWs. When measuring the PWV more locally, such as between the wrist and a position at the lower arm, the discrepancy between the distance between the sensors and the actual path length travelled by the PWs should diminish.

Furthermore, the utilized piezoelectric sensors in the Complior and PPG-sensors in the Biopac system were quite sensitive to motion and positioning disturbances. This sensitivity to disturbances poses a potential limitation on the usability of these techniques in clinical practice. Moreover, the Complior system is not always comfortable for the subject: use of the clip on the neck was sometimes experienced as uncomfortable, whereas the sensor required for the Biopac system a photoplethysmography sensor on the finger and three ECG-leads on the wrists and right ankle are more comfortable than the Complior sensor and are generally available in hospitals. Using this system could benefit patients and clinical practice. Although the ‘7Step PW-Filter’ algorithm used to eliminate distorted PWs functioned well, the availability of a PPG-sensor less sensitive to disturbances and not requiring measuring sensor distances, would greatly simplify measuring PWs in awake and moving patients. Obviously, this is less relevant when measuring PWs in patients under general anaesthesia.

In conclusion, this study demonstrated that PWV values were consistently and significantly lower when measured with the Biopac system than when measured with the Complior system. Yet, despite the difference in absolute PWV values, the two systems did agree fairly well. This suggests that as long as the difference in PWV magnitudes are taken into account, either system could be used to measure PWV changes in time. However, when basing diagnosis on absolute PWV values, one should be very much aware of how the PWV was measured and with what system. In the future, clinical practice could greatly benefit if software for calculating PWV is embedded in the commonly used anaesthesia monitors, enabling PWV measurements using a standard ECG and a standard pulse oximeter. This might allow PWV measurements to become a widely available diagnostic tool, and an easy-to-use, noninvasive, safe and quick method for objectively assessing arterial stiffness as a reliable prognostic parameter for cardiovascular morbidity and mortality.
